# Cellular and mitochondrial mechanisms of atrial fibrillation

**DOI:** 10.1007/s00395-020-00827-7

**Published:** 2020-11-30

**Authors:** Fleur E. Mason, Julius Ryan D. Pronto, Khaled Alhussini, Christoph Maack, Niels Voigt

**Affiliations:** 1 Institute of Pharmacology and Toxicology, University Medical Center Göttingen, Georg-August University Göttingen, Robert-Koch-Straße 40, 37075 Göttingen, Germany; 2 DZHK (German Center for Cardiovascular Research), Partner Site Göttingen, Göttingen, Germany; 3 Department of Thoracic and Cardiovascular Surgery, University Clinic Würzburg, Würzburg, Germany; 4 Department of Translational Research, Comprehensive Heart Failure Center Würzburg, University Clinic Würzburg, Am Schwarzenberg 15, 97078 Würzburg, Germany; 5 Department of Internal Medicine I, University Clinic Würzburg, Am Schwarzenberg 15, 97078 Würzburg, Germany; 6 Cluster of Excellence “Multiscale Bioimaging: From Molecular Machines to Networks of Excitable Cells” (MBExC), University of Göttingen, Göttingen, Germany

**Keywords:** Atrial fibrillation, Mitochondria, Electrophysiology, Oxidative stress, Calcium, Atrial cardiomyopathy

## Abstract

The molecular mechanisms underlying atrial fibrillation (AF), the most common form of arrhythmia, are poorly understood and therefore target-specific treatment options remain an unmet clinical need. Excitation–contraction coupling in cardiac myocytes requires high amounts of adenosine triphosphate (ATP), which is replenished by oxidative phosphorylation in mitochondria. Calcium (Ca^2+^) is a key regulator of mitochondrial function by stimulating the Krebs cycle, which produces nicotinamide adenine dinucleotide for ATP production at the electron transport chain and nicotinamide adenine dinucleotide phosphate for the elimination of reactive oxygen species (ROS). While it is now well established that mitochondrial dysfunction plays an important role in the pathophysiology of heart failure, this has been less investigated in atrial myocytes in AF. Considering the high prevalence of AF, investigating the role of mitochondria in this disease may guide the path towards new therapeutic targets. In this review, we discuss the importance of mitochondrial Ca^2+^ handling in regulating ATP production and mitochondrial ROS emission and how alterations, particularly in these aspects of mitochondrial activity, may play a role in AF. In addition to describing research advances, we highlight areas in which further studies are required to elucidate the role of mitochondria in AF.

**Electronic supplementary material:**

The online version of this article (10.1007/s00395-020-00827-7) contains supplementary material, which is available to authorized users.

## Introduction

Atrial fibrillation (AF) is the most common sustained arrhythmia and causes substantial morbidity and mortality, particularly due to embolic stroke [5, 88]. Currently available AF therapies have limited efficacy and safety, particularly in patients with long-standing persistent (‘chronic’) AF (cAF). A greater understanding of the molecular mechanisms promoting AF is expected to facilitate the development of improved and better-targeted anti-AF therapies [33, 48].

The underlying mechanisms initiating and promoting AF are incompletely resolved [81]. However, calcium (Ca^2+^) handling abnormalities and oxidative stress are thought to play central roles in the pathophysiology of AF. Mitochondria are the main producers of cellular adenosine triphosphate (ATP) in cardiac myocytes, and both Ca^2+^ and adenosine diphosphate (ADP) are key regulators of respiratory flux to match energy supply to the constantly varying demands in the heart [16, 63]. In mitochondria, the Krebs cycle is fuelled by products of glycolysis and fatty acid β-oxidation and produces nicotinamide adenine dinucleotide (NADH) and flavin adenine dinucleotide (FADH_2_), which donate electrons to complexes I and II of the electron transport chain (ETC), respectively. Through sequential redox reactions along the ETC, protons are translocated across the inner mitochondrial membrane, establishing a proton gradient which is harnessed by the *F*_1_/*F*_o_ ATP-synthase to phosphorylate ADP to ATP. During a physiological increase in workload, β-adrenergic stimulation increases the rate and amplitude of cytosolic Ca^2+^ transients which, on the one hand, increases ATP consumption. The ensuing increase in ADP flux to the ATPase drains electrons from NADH into the ETC (“pull conditions”), which per se would oxidise NADH. However, the increase in cytosolic Ca^2+^ transient amplitude and frequency increases Ca^2+^ uptake into mitochondria via the Ca^2+^ uniporter (MCU). Since mitochondrial Ca^2+^ uptake is fast, but extrusion is slower, heart (or myocyte) stimulation rate strongly controls mitochondrial Ca^2+^ accumulation [16].

**Fig. 1:**
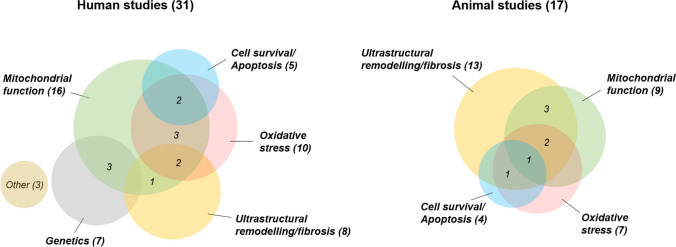
Results of a medical subject headings (MeSH) publication search in PubMed with categorisation. MeSH terms: “Atrial fibrillation” and “Mitochondria”. Relevant publications cited in the current review are also included. Review and editorial publications are excluded. Publications are listed in the Electronic Supplementary Material

Accumulation of Ca^2+^ subsequently increases the production of electron donors, the reduced forms of NADH and FADH_2_, used by the electron transport chain to produce ATP via oxidative phosphorylation. However, this “push condition” contributes to reactive oxygen species (ROS) production at the ETC which, when in excess, results in oxidative stress, as discussed later. Since oxidative stress is associated with AF and its risk factors, the mitochondria could be a potential upstream target for anti-AF therapeutics [110, 124, 125]. Extensive research on ventricular bioenergetics and the ensuing changes in heart failure has paved the way towards testing of mitochondria-targeted therapies, but this is not yet the case in AF [49]. Despite the obvious alterations in myocardial energy demand at increased stimulation frequency in AF, there is a scarcity of literature investigating how much of a role mitochondria play in the pathophysiology of AF. A Medical Subject Headings (MeSH) term search in PubMed, searching for “Atrial fibrillation” and “Mitochondria” yields very few results (Fig. 1 and Supplementary Material). In the first section of this review, we provide an overview of AF pathophysiology and general mitochondrial physiology before reviewing; in the second section, the existing literature associating mitochondrial dysfunction with AF. Furthermore, we discuss ideas how impaired mitochondrial function, especially with regard to Ca^2+^ handling, could contribute to a pro-arrhythmic substrate and we identify targets for future research.

## Pathophysiology of atrial fibrillation

**Fig. 2:**
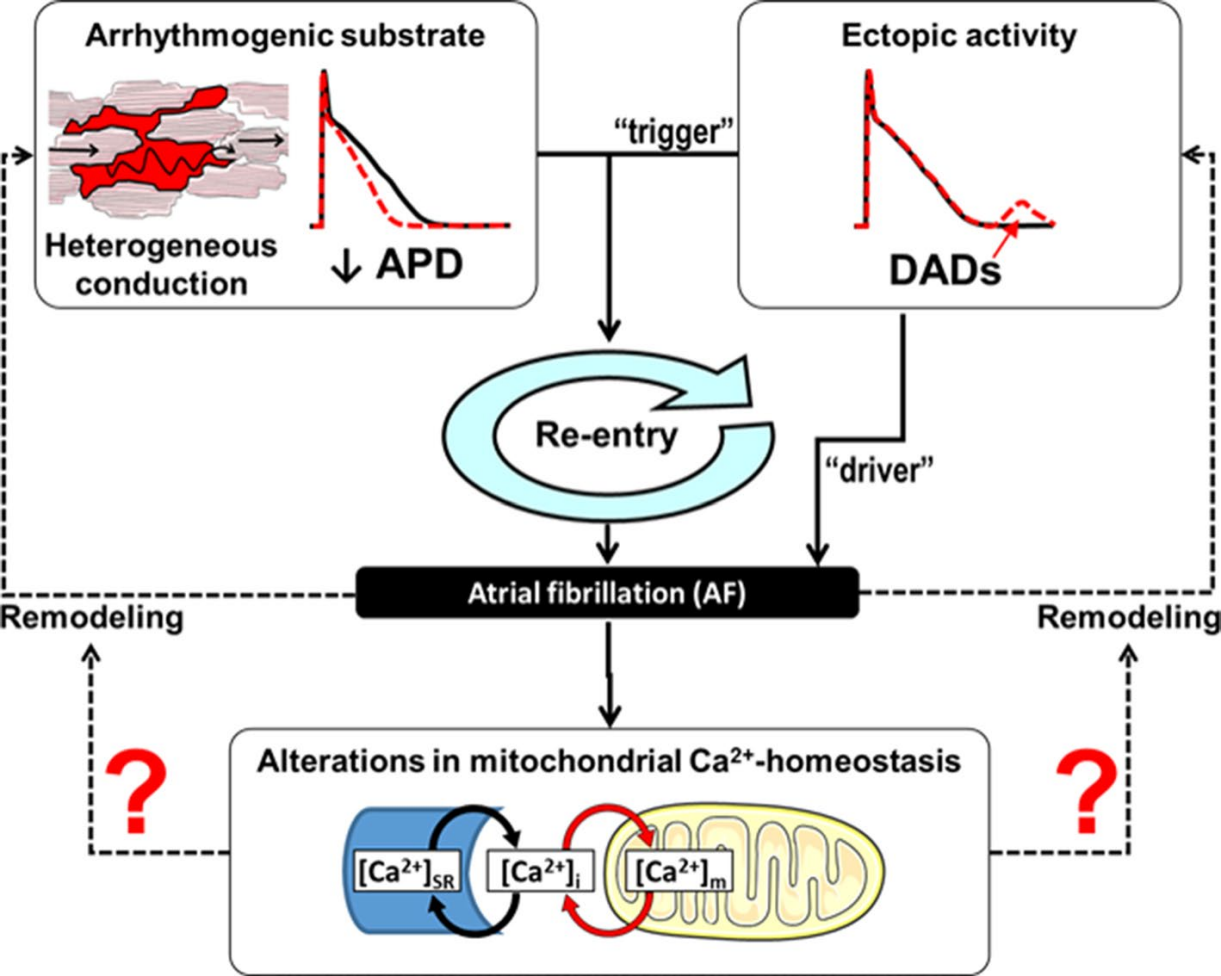
General mechanisms of atrial fibrillation and the potential involvement of disturbed mitochondrial Ca^2+^ handling. Re-entry requires a vulnerable substrate and trigger for initiation. Ectopic activity can maintain re-entry behaviour. APD, action potential duration; DAD, delayed afterdepolarisation (adapted from Heijman et al. [48])

The most accepted mechanisms underlying AF are re-entry and ectopic activity (Fig. 2) [49, 121, 129]. Re-entry describes a pathophysiological concept based on continued impulse propagation around a functional or structural obstacle. Occurrence of re-entry requires a vulnerable substrate and a trigger to initiate re-entry. AF is associated with electrical remodelling of various ion currents, resulting in re-entry-promoting shortening of atrial repolarisation, as previously reviewed [49]. This is exacerbated by fibrosis which is thought to play a central role in the pathophysiology of AF, providing a substrate for re-entry and its stabilisation [94]. Spatially discordant electrical alterations in excitability (alternans) cause electrical heterogeneity, favouring initiation and maintenance of AF. Ectopic activity describes abnormal impulse generation outside the sino-atrial node which can act as such a trigger for re-entry. Various publications, as recently reviewed by Denham et al., indicate that disturbances in intracellular Ca^2+^ handling may play a critical role in the development of ectopic activity, as discussed later [32]. Furthermore, ectopic activity, when occurring repetitively at high frequencies, can maintain the arrhythmia as a so-called “driver”. An example of abnormal impulse generation is triggered activity, such as delayed afterdepolarisations (DADs). Indeed, there is evidence in human cardiac myocytes implicating DADs as an underlying cause of both chronic and paroxysmal AF [117, 118].

### Abnormal Ca^2+^ handling contributes to initiation and maintenance of AF

Ca^2+^ enters cardiac myocytes through voltage-gated L-type Ca^2+^ channels (Ca^2+^ current, *I*_Ca,L_) and activates ryanodine receptors (RyR2) located in the sarcoplasmic reticulum (SR), triggering a much larger Ca^2+^ release than the initiating signal (Ca^2+^-induced Ca^2+^ release, CICR). The released Ca^2+^ binds to contractile proteins, causing contraction, and the entire process has been coined excitation–contraction coupling, as reviewed by Bers [15]. The subsequent removal of cytosolic Ca^2+^ ([Ca^2+^]_i_) allows diastolic relaxation and cardiac filling. Main Ca^2+^ removal mechanisms are the SR Ca^2+^-ATPase (SERCA2a), which pumps Ca^2+^ back into the SR, and the sodium-calcium exchanger (NCX1), which brings 3 Na^+^ ions into the cell per extruded Ca^2+^ ion. The extent to which mitochondrial Ca^2+^ uptake shapes the systolic Ca^2+^ transient remains somewhat controversial; however, the current consensus is that it has little effect on the free cytosolic Ca^2+^ concentration [16].

Pathological beat-to-beat alterations (alternans) in SR Ca^2+^ release can be translated into alterations of action potential duration (APD)—the aforementioned electrical alternans, as demonstrated in a sheep model by Pearman and colleagues [101]. This is presumably due to the close coupling of [Ca^2+^]_i_ with Ca^2+^-dependent ion channels and transporters such as L-type Ca^2+^ channel and NCX1. [Ca^2+^]_i_-driven alternans is enhanced by factors increasing SR Ca^2+^ release and by factors reducing Ca^2+^ sequestration from the cytosol, such as increased SR Ca^2+^ leak and reduced SERCA expression or activity (for example due to reduced ATP levels) [34, 37]. Since electrical alternans has been linked to metabolic oscillations, it is plausible that mitochondrial dysfunction-related alternans may also contribute to arrhythmogenesis in AF [98]. Such oscillations in myocytes and whole heart depend on increased levels of ROS, as discussed later in further detail [2, 7, 29].

Increased open probability of RyR2 predisposes to spontaneous (non-AP-triggered) diastolic SR Ca^2+^ release events (SCaEs) [27, 52, 95, 118]. CaMKII-mediated hyperphosphorylation of RyR2 contributes to RyR2 dysfunction in AF [50, 118]. Greater intracellular Ca^2+^ leak, together with increased NCX1 function, promotes the aforementioned DADs, which have the potential to trigger extrasystolic activity [118]. Intracellular Ca^2+^ overload, secondary to intracellular Na^+^ overload, also leads to this type of activity, as previously reviewed [80].

## Mitochondrial physiology in the heart

### Mitochondrial Ca^2+^ homeostasis in cardiac myocytes

The importance of Ca^2+^ in the mitochondrial matrix ([Ca^2+^]_m_) for regulation of mitochondrial function was suggested as far back as 30 years ago [83]. Although the kinetics of mitochondrial Ca^2+^ cycling and contribution to cytosolic [Ca^2+^] remain highly controversial, it is well accepted that Ca^2+^ regulates ATP production and ROS signalling and plays a crucial role in mitochondrial-determined cell death [16]. Furthermore, the coupling between cytosolic and mitochondrial Ca^2+^ increases ATP synthesis in response to increased cardiac activity, thus matching cellular ATP supply to demand [59]. Considering that Ca^2+^ is important in the regulation of mitochondrial function, it is conceivable that altered cytosolic Ca^2+^ dynamics may affect mitochondrial Ca^2+^ handling and energetics (Fig. 2). While the majority of previous studies on cardiac mitochondria have been conducted in ventricular myocytes or tissue, it has been revealed that mitochondria may buffer centripetal diffusion of Ca^2+^ in atrial myocytes and there is evidence that mitochondrial distribution and density vary between atrial and ventricular tissue [20, 77, 84]. Therefore, atrial-specific properties of mitochondria is an area warranting further investigation. Furthermore, since mitochondrial dysfunction has been implicated in heart failure, it will be important to ascertain whether—or how—mitochondria are associated with atrial pathological processes, such as those occurring in AF.

#### Kinetics of mitochondrial Ca^2+^ transients

**Fig. 3:**
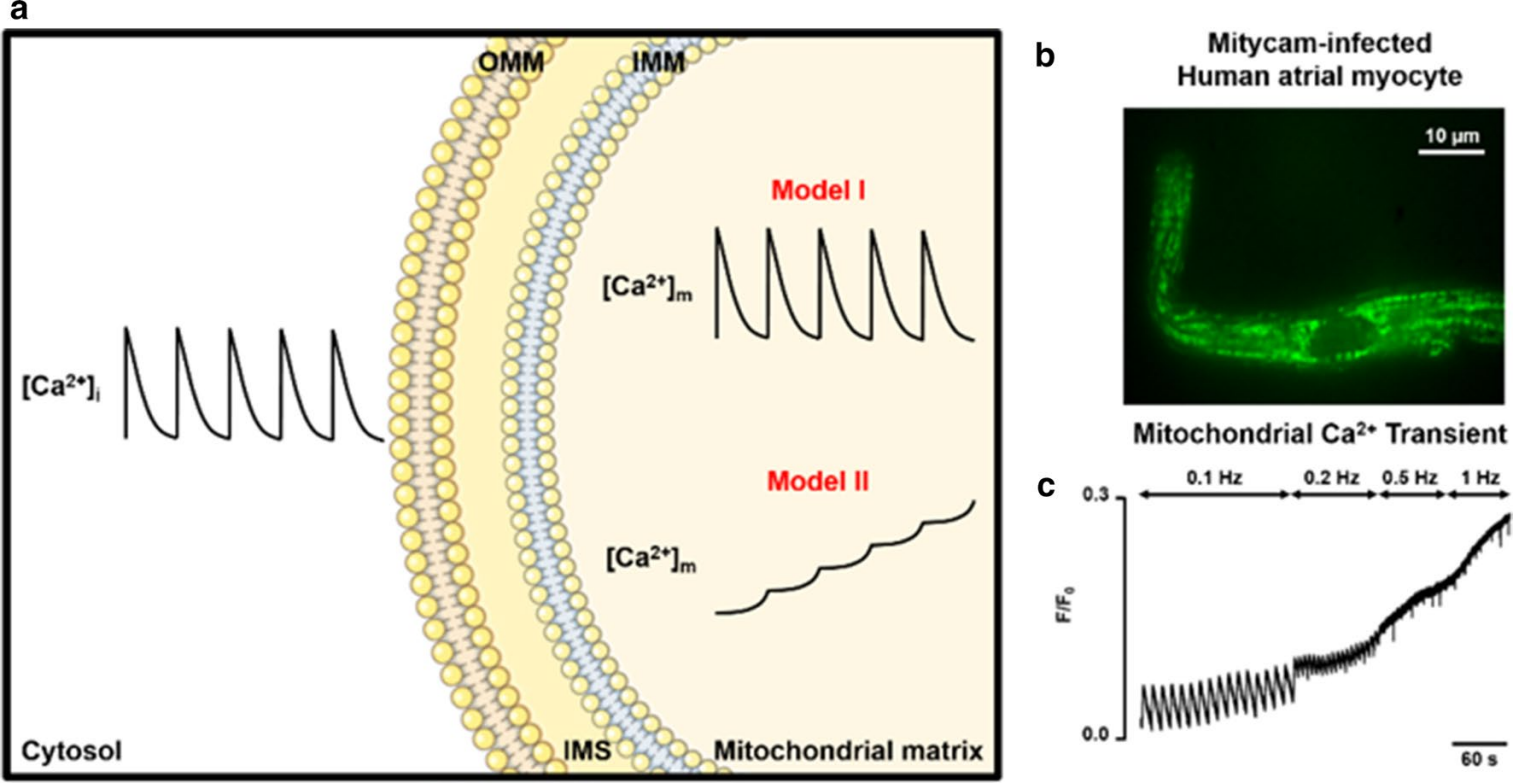
Dynamics of mitochondrial Ca^2+^. **a** Models of transmission of fast cytosolic Ca^2+^ transients ([Ca^2+^]_i_) to mitochondrial Ca^2+^ ([Ca^2+^]_m_). Model I: rapid, beat-to-beat transmission; Model II: slow integration of [Ca^2+^]_i_ oscillations. IMM, inner mitochondrial membrane; IMS, intermembrane space; OMM, outer mitochondrial membrane. **b** Fluorescent image of a Mitycam-infected human atrial myocyte (measurement of [Ca^2+^]_m_.) c) Representative recording of Mitycam fluorescence in response to increasing stimulation frequency in a human atrial myocyte (unpublished). This figure was created using images from Servier Medical Art Commons Attribution 3.0 Unported License. (http://smart.servier.com). Servier Medical Art by Servier is licensed under a Creative Commons Attribution 3.0 Unported License

The kinetics of mitochondrial Ca^2+^ uptake are still a matter of debate. It has been suggested that mitochondria take up and release Ca^2+^ during each heart beat (Fig. 3a, Model I: Oscillator) or that they may act as integrators, taking up Ca^2+^ gradually in response to increased cytosolic Ca^2+^, resulting in an increase in steady state [Ca^2+^]_m_ (Fig. 3a, Model II: Integrator) [75]. Difficulties in measuring [Ca^2+^]_m_ with membrane-permeable fluorescent ester indicators likely contribute to the uncertainty as to which model is correct [75]. There is, however, evidence for both models in combination (Fig. 3b, c) [6, 69, 74]. It appears that [Ca^2+^]_m_ transients can occur with each cytosolic Ca^2+^ transient, and that diastolic [Ca^2+^]_m_ increases progressively at higher stimulation frequencies or cellular Ca^2+^ loading. Whereas the oscillator model seems to be relevant at lower stimulation frequencies, the integrator model is thought to dominate at higher frequencies, which is important for matching ATP production to demand. Moreover, peak Ca^2+^ uptake in the mitochondria appears to occur faster than cytosolic Ca^2+^ increase, supporting the concept of a Ca^2+^ microdomain between the SR and mitochondria that is necessary for efficient Ca^2+^ uptake [58, 74].

#### Mitochondrial Ca^2+^-influx and -efflux pathways

**Fig. 4:**
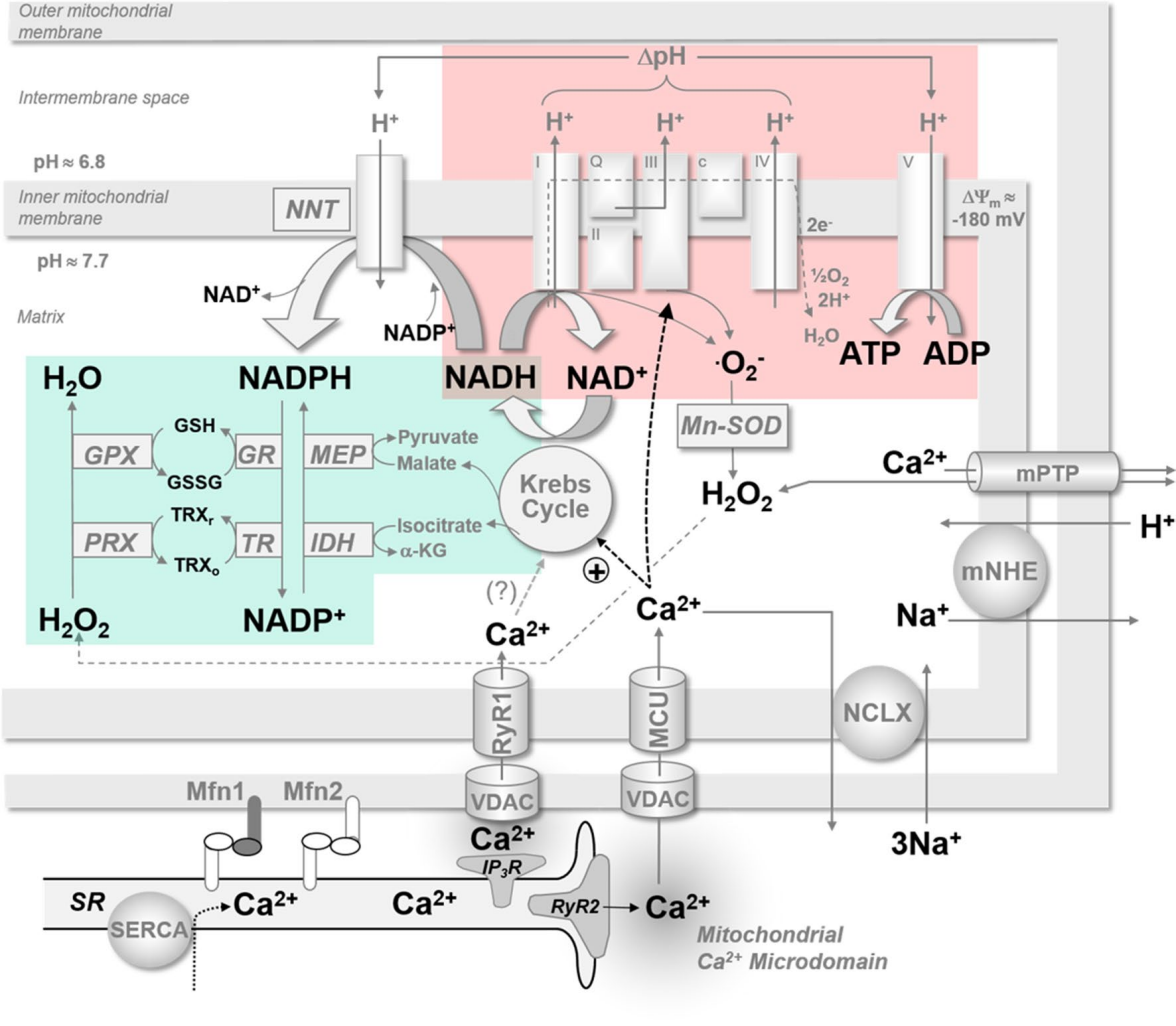
Major mitochondrial Ca^2+^-influx and -efflux pathways, mitochondrial ATP production and ROS elimination. Ca^2+^ is released by the sarcoplasmic reticulum (SR) via type 2 ryanodine receptors (RyR2) and passes through the outer mitochondrial membrane via voltage-dependent anion channels (VDAC). Ca^2+^ enters the mitochondrial matrix via the mitochondrial Ca^2+^ uniporter (MCU) in the inner mitochondrial membrane. Mitochondrial ryanodine receptor type 1 (RyR1) may play a role in taking up Ca^2+^ released more slowly from the SR via inositol 1,4,5-triphosphate receptors (IP_3_R). Microdomains of high [Ca^2+^] are created due to the close proximity of SR and mitochondria through membrane tethering by mitofusin 1 and 2 (Mfn1 and Mfn2). Mitochondrial Ca^2+^ is extruded on the Na^+^/Ca^2+^/Li^+^ exchanger (NCLX). The mitochondrial permeability transmission pore (mPTP) opens upon Ca^2+^ overload and plays a role in cell death and ROS-induced ROS release (RIRR). Matrix Ca^2+^ activates Krebs cycle dehydrogenases, regenerating the reduced form of NADH (nicotinamide adenine dinucleotide) which donates electrons to the electron transport chain (ETC). Electron flow in the ETC causes protons to be translocated into the intermembrane space, contributing to an electrochemical gradient across the inner mitochondrial membrane (∆*Ψ*_m_) which is used to drive ATP production by the *F*_1_/*F*_o_ ATP-synthase (Complex V). Complexes I and III of the ETC produce superoxide (^∙^O_2_^−^) which is subsequently converted to H_2_O_2_ by superoxide dismutase (Mn-SOD). H_2_O_2_ is eliminated by peroxiredoxin (PRX) and glutathione peroxidase (GPX), which require reduced NADPH (nicotinamide adenine dinucleotide phosphate) for regeneration. NADPH is regenerated by isocitrate dehydrogenase (IDH) and malic enzyme (MEP) and nicotinamide nucleotide transhydrogenase (NNT). *α-KG* α-ketoglutarate, *I–IV* complexes I–IV of the ETC, *Q* Q-cycle, *ΔpH* proton gradient; mNHE, mitochondrial Na^+^–H^+^ exchanger, *SERCA* SR Ca^2+^-ATPase, *GSH/GSSG* reduced/oxidised glutathione, *GR* glutathione reductase, *TRX*_*r*_*/TRX*_*o*_ reduced/oxidised thioredoxin, *TR* thioredoxin reductase (adapted from Nickel et al. [96])

The major mitochondrial Ca^2+^-influx and -efflux pathways are summarised in Fig. 4. Mitochondrial Ca^2+^ microdomains necessary for Ca^2+^ influx are spatially distinct from Ca^2+^ efflux mechanisms, and this is likely to be important for efficient Ca^2+^ signalling and excitation-energetics coupling [62]. The mitochondrial matrix is separated from the cytosol by two membranes which need to be passed for exchange of metabolites, adenine nucleotides and cations, including regulators of mitochondrial activity such as Ca^2+^. A voltage-dependent anion channel (VDAC) allows Ca^2+^ to pass across the outer mitochondrial membrane, and thus plays a role in mediating mitochondrial Ca^2+^ uptake [104, 123]. The relative low permeability of the inner mitochondrial membrane (IMM) to ions is required to maintain the strongly negative membrane potential between the intermembrane space and the mitochondrial matrix (∆*Ψ*_m_ ≈ − 180 mV). Ions can only pass into the mitochondrial matrix via specialised channels and transporters. Several mechanisms have been described for mitochondrial Ca^2+^ uptake, all driven by the large ∆*Ψ*_m_ across the IMM. The best-characterised pathway is the mitochondrial Ca^2+^ uniporter (MCU) [61, 112]. The MCU is regulated by divalent cations, ruthenium compounds and adenine nucleotides. The Ca^2+^-binding protein MICU1 (mitochondrial Ca^2+^ uptake 1) is likely required for mitochondrial Ca^2+^ uptake and may act as an auxiliary regulatory protein, determining the [Ca^2+^]_m_ threshold for Ca^2+^ uptake and preventing mitochondrial Ca^2+^ overload. Low Ca^2+^ affinity (*K*_m_ ≈ 10–20 µM) is a critical property of the MCU, making mitochondrial proximity to microdomains of high [Ca^2+^]_i_ (e.g. RyR2) a requirement for effective mitochondrial Ca^2+^ uptake. The importance of this proximity has been demonstrated by Lu and colleagues in rabbit ventricular myocytes transfected with a genetically encoded mitochondrial Ca^2+^ sensor (Mitycam); it was found that mitochondrial Ca^2+^ uptake is greater and faster near SR Ca^2+^ release sites (Z-line of sarcomere), compared to the middle of the sarcomere (M-line), where SR Ca^2+^ release is limited and MCU density is lower [69]. The role of the mitochondrial MCU complex during basal metabolism versus stress conditions has been extensively discussed by others but the exact involvement of the MCU during pathophysiology remains to be elucidated [41, 47].

Two other potential avenues of Ca^2+^ entry into mitochondria are rapid-mode mitochondrial Ca^2+^ uptake (RaM) and mitochondrial RyRs (mRyR1), as reviewed by De Stefani et al. [113]. RaM was found in heart mitochondria after its initial discovery in liver [23]. However, RaM is thought to be negligible during physiological [Ca^2+^]_i_ changes in cardiac myocytes because of its slow recovery from inactivation. In contrast, mRyR1 allows Ca^2+^ sequestration at low cytosolic Ca^2+^ concentrations and therefore it could contribute to physiological beat-to-beat Ca^2+^ uptake in the heart [17]. Furthermore, there are data showing that mitochondrial RyR1 may also be important in slower uptake of inositol 1,4,5-triphosphate receptor (IP_3_R)-mediated SR Ca^2+^ release [106, 107].

Mitochondrial Ca^2+^ extrusion pathways comprise both Na^+^-dependent and Na^+^-independent mechanisms. However, in cardiac mitochondria, the mitochondrial Na^+^–Ca^2+^ exchanger (mNCX or NCLX [Na^+^/Ca^2+^/Li^+^ exchanger]) is suggested to be the predominant Ca^2+^ extrusion mechanism [24, 100]. Moreover, lethality observed in NCLX-KO mice was attributed to mitochondrial Ca^2+^ overload, which consequently results in cardiac remodelling and dysfunction [71]. It is widely accepted that NCLX is electrogenic and exchanges 3 Na^+^ ions per extruded Ca^2+^ ion, favouring Ca^2+^ extrusion due to the highly negative ∆*Ψ*_m_ [90]. To maintain intra-mitochondrial Na^+^ concentration, Na^+^ is removed from the mitochondrial matrix by a Na^+^–H^+^ exchanger (mNHE) located in the IMM. Of note, NCLX develops half-maximal activity in the physiological range of cytosolic Na^+^, making NCLX sensitive to physiological Na^+^ fluctuations and the cytosol-matrix Na^+^ gradient, as previously reviewed [14, 16, 90, 122].

### Mitochondrial Ca^2+^ regulates mitochondrial function

#### Mitochondrial ATP synthesis

It has been shown in non-ischaemic ventricular myocytes that almost all ATP is derived from mitochondrial oxidative phosphorylation, with the remainder from glycolysis and GTP formation in the Krebs cycle. During oxidative phosphorylation, four multi-protein complexes (Complexes I–IV) which make up the aforementioned ETC, located in the IMM, catalyse electron transport from NADH and FADH_2_ to oxygen. The energy released by electron transport allows Complexes I, III and IV to pump protons from the mitochondrial matrix into the intermembrane space; the resulting proton gradient across the IMM contributes to the electrochemical gradient (∆*Ψ*_m_), which fuels ATP synthesis by *F*_1_/*F*_o_ ATP-synthase (also known as Complex V) (Fig. 4).

It is thought that there is a complete turnover of the myocardial ATP pool in less than a minute under normal conditions and, depending on heart rate, this may be as quick as every 10 s [111]. Therefore, there is need for tight regulation of mitochondrial ATP production to meet varying metabolic demands resulting from beat-to-beat changes in cardiac workload. Two major regulators of oxidative phosphorylation have been proposed: (1) the products of ATP hydrolysis itself (ADP and P_i_) and (2) Ca^2+^. The classical hypothesis was based on measurements of oxygen consumption in isolated mitochondria [64]. ADP and *P*_i_ directly stimulate the *F*_1_/*F*_o_-ATP synthase, thus acting as a “pull” on oxidative phosphorylation and causing NADH oxidation. This hypothesis, however, has been challenged by the finding that large changes in cardiac work and ATP consumption in vivo are not associated with measurable changes in ADP/*P*_i_ [9]. An increased workload requires increased time-averaged [Ca^2+^]_i_. This also acts to increase [Ca^2+^]_m_. [Ca^2+^]_m_ stimulates substrate flow through the Krebs cycle by directly activating key enzymes, thereby increasing production of NADH and FADH_2_, fuelling oxidative phosphorylation and increasing ATP production [18, 58]. In addition, mitochondrial Ca^2+^ is thought to directly stimulate the *F*_1_/*F*_o_ ATP-synthase and possibly Complex III of the ETC, thus increasing the rate of oxidative phosphorylation [89, 114]. IP_3_R, activated by endothelin signalling, may also play an important role in the bioenergetics of cardiac myocytes by inducing mRyR1-mediated Ca^2+^ entry and consequently stimulating mitochondrial ATP production. This is thought to be due to close interaction between the SR and mitochondria, facilitated by mitofusin 2 (Mfn2) [106, 107].

#### Mitochondrial ROS production and elimination

Reactive oxygen species (ROS) in the mitochondria result from the single-electron reduction of molecular oxygen (O_2_) during oxidative phosphorylation, among other less significant pathways [91]. Since O_2_ is preferentially partitioned in biological membranes where it can interact with electron carriers, such as in the ETC, mitochondria are considered to be a major source of ROS in the cell [10, 91]. Experimentally, 1–2% of oxygen consumed ends up in ROS production at the ETC, primarily at Complexes I and III, although this is estimated to be considerably less in vivo [91]. The first product of the monovalent reduction of O_2_ is superoxide anion (^·^O_2_^−^). Complex I produces ^·^O_2_^−^ by two mechanisms: (1) a high NADH/NAD^+^ ratio results in fully reduced flavin mononucleotide (FMN), thus backing up the electrons, increasing the time for interaction with O_2_; (2) reverse electron transport (RET) due to a reduced ubiquinone (CoQ) pool, where the rate of ^·^O_2_^−^ production is considered to be highest in the mitochondria [91]. Complex III releases ^·^O_2_^−^ at least partially into the intermembrane space, depending on local ubisemiquinone (Q^·−^) concentrations in the *bc*_*1*_ complex, whereas ROS production and release by Complex I is limited to the matrix [19]. ROS is generated at Complex III via the Q-cycle when, in a reduced state, a halt in the electron flow enables more time for O_2_ to interact with the reduced electron carrier, Q^·−^ [116]. ROS production at Complex III, however, is regarded as inconsequential compared to rate of production at Complex I, unless pharmacologically induced by Antimycin A. Dihidrolipoamide dehydrogenase, a component of the metabolic enzymes α-ketoglutarate dehydrogenase (αKGDH) and pyruvate dehydrogenase (PDH), is also capable of ROS production in a NADH/NAD^+^-dependent manner [3, 91, 103]. However, under physiological conditions in working cardiac myocytes, α-KGDH is not a relevant source of ROS, but rather contributes to regeneration of reduced nicotinamide adenine dinucleotide phosphate (NADPH) through tight functional coupling to the nicotinamide nucleotide transhydrogenase [119]. Further potential sites for ROS production in the mitochondria have been suggested, as reviewed by Murphy [91]. ROS production increases when there is low ATP demand (state 4 respiration), causing a build-up of reduced NADH (electron donors) or when there is damage to the ETC [10]. Furthermore, ROS production in cardiac myocytes rises during increased workload, for example at higher stimulation frequencies [51, 56].

Net mitochondrial ROS emission from mitochondria is determined not only by the ROS formation rate, but also by ROS elimination. ^∙^O_2_^−^ is assumed to be present in low picomolar range due to its immediate dismutation by MnSOD [60, 91]. H_2_O_2_, the main ROS signal, is eliminated by peroxiredoxin and glutathione peroxidase, which require NADPH for regeneration, and also by mitochondrial catalase [16]. NADH generated by the Krebs cycle, and in particular, by α-KGDH, is converted to NADPH by nicotinamide nucleotide transhydrogenase (NNT), a process coupled to the proton gradient across the IMM [97, 119]. Furthermore, NADPH is regenerated by isocitrate dehydrogenase and malic enzyme, which also both derive substrates from the Krebs cycle (isocitrate and malate, respectively). Thus mitochondrial anti-oxidative capacity, ROS elimination and *net* mitochondrial ROS emission are largely dependent on the Krebs cycle turnover rate [16, 59]. Increased mitochondrial Ca^2+^ uptake and consecutively, enhanced Krebs cycle turnover rate maintain sufficient anti-oxidative capacity of the mitochondrial matrix during increased workload. Accordingly, myocytes from a heart failure model, in which [Ca^2+^]_m_ elevation in response to increased workload is limited, showed abnormal increases in mitochondrial ROS emission at higher stimulation frequencies [56].

The group of O’Rourke introduced the idea of redox-optimised ROS balance (R-ORB) [8, 30]. R-ORB conceptualises that the redox environment (RE; calculated from the oxidised and reduced states of mitochondrial redox couples) determines mitochondrial ROS levels, and that an intermediate redox state (maximal energy output, state 3 respiration) is accompanied by minimal ROS emission. At either extreme of the RE, ROS levels increase, albeit through different mechanisms; at extremely reduced RE, ROS formation excels due to increased electron slippage from the ETC and thus ROS production exceeds ROS scavenging. Conversely, in the case of an oxidative shift in the RE, for example during pathological increase in workload during heart failure, ROS emission will increase due to reduced ROS-scavenging capacity of the mitochondria, secondary to NNT reversal, as discussed later [97].

#### Mitochondrial Ca^2+^ overload

Mitochondrial Ca^2+^ overload leads to pathological opening of the mitochondrial permeability transition pore (mPTP), causing a profound decrease in mitochondrial membrane potential and ATP levels which leads to cell death [31, 44]. Interestingly, small, transient and low conductance openings of the PTP (tPTP) have also been identified; however, such openings are believed to be rare and are thought to occur under *physiological* conditions as a way to release excess mitochondrial Ca^2+^ [70]. The major role of the mPTP in cell death has been reviewed comprehensively elsewhere and will therefore not be the focus of this review [31, 44].

## Mitochondria and atrial fibrillation

### Ultrastructural remodelling in cardiac pathophysiology

Subcellular anatomy is thought to be crucial for effective mitochondrial Ca^2+^ uptake, which requires close interaction between MCU and RyR2 of the SR, as mentioned earlier. In ventricular myocytes, mitochondria are located ~ 40 to 300 nm from the RyR2 and are, therefore, exposed to very high [Ca^2+^]_i_ (10-20 µM), explaining why mitochondrial Ca^2+^ uptake through MCU is so rapid, despite its relatively low Ca^2+^ affinity [109]. Thus, as has been observed in ventricular myocytes, mitochondrial Ca^2+^ uptake efficiency is maximal when Ca^2+^ is released from the SR, compared to Ca^2+^ entering the cell via sarcolemmal NCX [57]. This points to RyR2 channels as the major supplier of Ca^2+^ for the mitochondria and suggests that SR and mitochondria must have close proximity. Molecular tethers such as Mfn2 likely maintain the close structural and functional association between SR and mitochondria. Suppression of Mfn2 in murine embryonic fibroblasts and HeLa cells increases the separation between SR and mitochondria, although this has spurred some controversies [21, 39, 92, 93]. Furthermore, mitochondrial Ca^2+^ uptake is decreased in ventricular myocytes from Mfn2-deficient mice [26, 107].

The spatial organisation of mitochondria is disrupted in heart failure [42, 72] and even as early as the 1980s, the Rosen group observed that ultrastructure of *atrial* myocytes can also be damaged in response to different cardiac and metabolic diseases, potentially impacting on RyR-mitochondrion interaction [79]. These findings have been supported by a more recent publication, showing that ultrastructural remodelling occurs in the left atrium of patients with AF [108]. Furthermore, outer mitochondrial membrane disruption has been observed in an AF mouse model with constitutively leaky RyR2, and this may affect physical tethering between the mitochondria and SR [124]. There is also evidence for a RyR2-/VDAC2-containing protein complex, which may play a role in stabilising SR-mitochondrial interaction and which seems to be essential for the transfer of Ca^2+^ from the SR to the mitochondrial matrix, at least in ventricular cardiac myocytes [38, 86]. The IP_3_R is thought to play a greater role in the physiology of the atrium than the ventricle and has been shown to contribute to endothelin-induced arrhythmic activity in a rat model [68, 76]. Whether IP_3_R-related pathological mechanisms induce or involve mitochondrial dysfunction remains unknown.

#### Open aspects for future research

The various interactions between intracellular Ca^2+^ release sites and mitochondria, to what extent they are altered in AF and the effects of such changes on mitochondrial Ca^2+^ uptake remain unclear and therefore represent major targets for future investigation. Previous studies on mitochondrial activity in ventricular cells/tissue will undoubtedly serve as an invaluable basis for the design of atrial studies [56, 69]. Measurement of atrial mitochondrial (and cytosolic) Ca^2+^ at high pacing frequencies, as well as MCU function, is likely to be a crucial step in ascertaining the roles of Ca^2+^ handling dynamics between cytosolic and mitochondrial compartments and whether any alterations occur during—and contribute to—AF pathophysiology.

### The potential role of impaired mitochondrial ATP synthesis in atrial fibrillation

As mentioned previously, the majority of cellular ATP is produced by oxidative phosphorylation in the mitochondria. Emelyanova and colleagues provided evidence for impaired activity of Complexes I and II in right atrial tissue from patients with AF [35]. These findings are in agreement with another recent study which observed impaired complex I and II function in addition to impaired ETC super**-**complex assembly in patients with diabetes and AF, compared with diabetes alone [54]. Down-regulated expression of various enzymes involved in mitochondrial energy metabolism, for example citrate synthase, has been identified in AF [115]. There is also evidence for pre-operative downregulation of mitochondrial/oxidative phosphorylation gene clusters as well as mitochondrial dysfunction in patients who develop AF after cardiac surgery (post-operative AF) [1, 87]. Animal models of AF have yielded disparate results, for example reduced Complex III and *F*_1_/*F*_o_ ATP-synthase activity in a canine model of AF [78]. Therefore, further investigation into human AF will add to the current knowledge about changes occurring at the level of the ETC and mitochondrial respiration during the disease process.

**Fig. 5:**
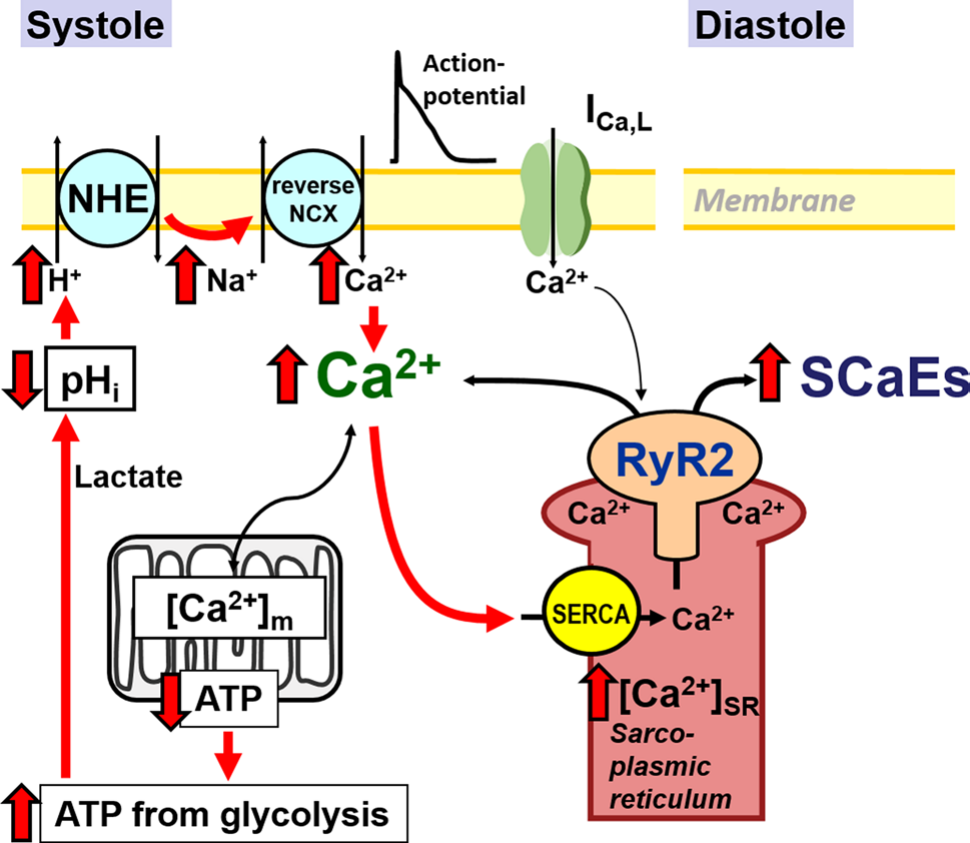
The impact of reducing mitochondrial ATP production. Compensatory increase in glycolysis reduces intracellular pH, consequently causing intracellular Na^+^ and Ca^2+^ overload (as suggested by Zima et al. [128]). *I*_*Ca.L*_ L-type Ca^2+^ current, *NCX* sodium–calcium exchanger, *NHE* sodium–hydrogen exchanger, *RyR2* ryanodine receptor type 2, *SCaEs* spontaneous Ca^2+^ release events, *SERCA* SR Ca^2+^-ATPase

The Blatter group used pharmacological tools to investigate the effect of impaired mitochondrial ATP synthesis in cat atrial myocytes [128]; cytosolic ATP concentration was unchanged, and it was deduced that increased glycolysis in the cytosol could compensate for lower mitochondrial ATP synthesis. However, there was also an increased frequency of the aforementioned SCaEs, as well as intracellular acidosis, and the authors suggested that increased lactate production (due to glycolysis) leads to acidosis, followed by Na^+^ overload via sarcolemmal Na^+^-H^+^ exchanger (NHE) and subsequent Ca^2+^ overload (via reverse-mode NCX), leading to SCaEs, as illustrated in **Fig.** 5 [128]. As mentioned previously, alterations of intracellular Ca^2+^ handling are thought to play a central role in the initiation and maintenance of arrhythmic activity, and, therefore, the findings of the Blatter group provide an attractive theory as to how altered mitochondrial function could provide a pro-arrhythmic substrate.

Due to the high stimulation frequency endured during AF, the energy consumption and, therefore, energy requirement of atrial tissue is increased, likely activating the aforementioned “pull” condition at the ETC [59]. It is believed that there are changes affecting supply and delivery of metabolic substrates and oxygen, inducing a state of metabolic stress [45]. We speculate that impaired cytosolic-mitochondrial Ca^2+^ signalling could also play a role. The initial phase of AF has been described as a short period of “cellular Ca^2+^ overload” on account of increased atrial activation rate, preceding a longer phase involving adaptation and remodelling of electrophysiology and Ca^2+^ dynamics, coined “Ca^2+^ silencing”, as reviewed by Greiser and Schotten [43]. It is thus plausible that a relative reduction of intracellular Ca^2+^, after the initial overload period, may contribute to the mismatch of ATP supply to demand in AF, considering the important role of Ca^2+^ in stimulating mitochondrial ATP production. It may not be the amount of Ca^2+^ per se entering the mitochondria which is important, but the amount relative to what is required for an increase in ATP production, for the energetic demands of the myocyte to be met.

Of course, many factors determine ATP production and there is increasing evidence that metabolic alterations play a crucial role in cardiac remodelling leading to arrhythmia, including AF, as comprehensively discussed by others [12, 13, 45, 99]. An extensive discussion of the metabolic alterations linked to AF is beyond the scope of this review. Nevertheless, a metabolic shift to a more fetal phenotype, i.e. from fatty acid β-oxidation to glycolysis, is thought to occur in permanent AF, thereby increasing the ratio of ATP produced per used O_2_ [12, 45]. Furthermore, increased utilisation of ketone bodies has been reported in AF [82]. Importantly, there is growing evidence suggesting that metabolic substrate usage is dependent on the stage of AF; adenosine monophospahte-activated protein kinase (AMPK), an energy sensor and regulator of several pathways, is thought to protect against metabolic stress by improving both mitochondrial function (control of glycolysis vs. fatty acid β-oxidation) and intracellular Ca^2+^ handling [25, 46, 66]. It has been suggested that AMPK activation, and thus its protective effects, occur during paroxysmal AF, but that this phenomenon is lost when AF becomes persistent [46].

#### Open aspects for future research

**Fig. 6:**
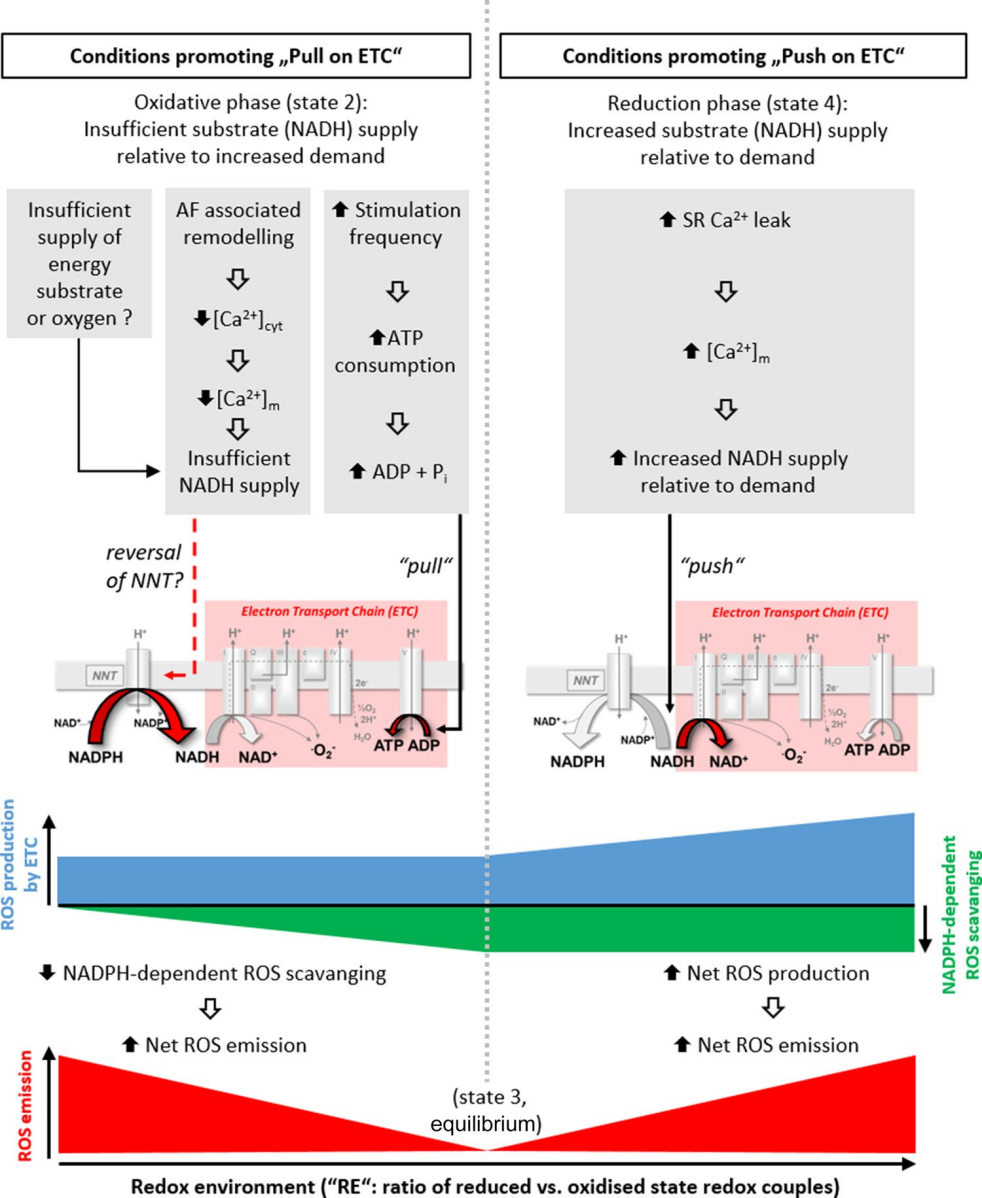
Hypothesis of net ROS production during atrial fibrillation with a focus on mitochondrial Ca^2+^ handling. ATP requirement is increased during atrial fibrillation (due to increased workload), causing a “pull” on the electron transport chain (ETC) (left). Due to remodelling, there is inadequate mitochondrial Ca^2+^ ([Ca^2+^]_m_) to sufficiently increase ATP production, e.g. NADH (nicotinamide adenine dinucleotide) production by the Krebs Cycle. NADPH (nicotinamide adenine dinucleotide phosphate) is converted to NADH by reverse mode NNT (nicotinamide nucleotide transhydrogenase) as a compensatory mechanism, at the expense of NADPH-driven ROS scavenging. Conversely, increased SR Ca^2+^ leak (right) could expose mitochondria to high Ca^2+^, thereby creating a “push” on the ETC and increasing mitochondrial ROS production such that it exceeds mitochondrial ROS scavenging capacity

It will be important in future investigations to identify the weakest link(s), i.e. the “limiting factor”, in matching ATP supply to demand in long-term AF. Energetic substrate- and oxygen supply, metabolism-related enzyme activity and ETC activity all represent potential culprits, with aspects of the latter two being partially under Ca^2+^-controlled regulation, as discussed earlier. We suggest that the “limiting factor” may depend on the extent of electrophysiological and structural remodelling and, therefore, the stage of AF. In response to pathological elevations of cardiac workload in the mouse, the mitochondrial transhydrogenase (NNT) is able to support ATP production by reversing its direction, i.e. converting NADPH to NADH, the conformational aspects of which have recently been defined [53, 97]. This “compensatory” mechanism, however, is at the expense of mitochondrial ROS scavenging and thus net mitochondrial ROS emission increases. Whether a similar situation occurs in chronic AF, due to increased and unmet energy requirements, is currently unknown, but this represents a potentially important avenue of research for the future (Fig. 6). Measurements of mitochondrial membrane potential should be included in future investigations, as it has been suggested that initial Ca^2+^ overload conditions alter the potential, thereby leading to impaired ATP production [67].

### The potential role of ROS in atrial fibrillation

An association between oxidative damage and AF in human tissue was first provided by Mihm and colleagues and it is now widely accepted that AF is associated with increased oxidative stress [35, 67, 85]. Although there are many pathways that produce ROS in mammalian cells, four major enzymatic systems seem to dominate in cardiac myocytes: mitochondria, NADPH oxidase, uncoupled NO synthase, and xanthine oxidase [16, 91]. It is believed that atrial remodelling during AF leads to a switch of ROS sources, from NADPH oxidase in early stages to mitochondrial- and eNOS-derived ROS in chronic AF [105]. Furthermore, mitochondrial ROS have been implicated in cardiac fibrosis, which is a hallmark of AF [40]. There is evidence suggesting that mitochondria play an important role in redox imbalance in post-operative AF, where increased levels of ROS and MnSOD activity, as well as increased sensitivity to mPTP opening, were observed [4, 87]. Monoamine oxidase in the outer mitochondrial membrane produces H_2_O_2_, and an increase in its activity may be a potential predictive marker for poAF [4]. A decrease in antioxidant-related gene expression and an increase in ROS-related gene expression have been observed in patients with permanent AF [55]. ROS production-elimination imbalance in AF leads to morphological and functional changes in the affected human cardiac myocytes, leading to an oxidative vicious cycle [67]. For example, there is evidence that mitochondrial DNA deletion and damage (mtDNA lesions) occurs in human AF [67]. Initial Ca^2+^ overload and chronic high oxidative stress levels in fibrillating atria may explain the rapid damage of mtDNA.

Atrial fibrillation occurs increasingly with age [65], and ageing results in metabolically dysfunctional cells due to mtDNA deletions [11]. This results in a “mosaic” pattern of dysfunctional cells in the cardiac tissue of mice, causing a pro-arrhythmic substrate [11]. A rapid pacing investigation by Bukowska and colleagues linked tachycardia and tachyarrhythmia with mitochondrial dysfunction and oxidative stress [22]. Furthermore, the Marks group suggested a role of ROS in atrial arrhythmogenesis: mice with a phosphomimetic mutation of RyR2 exhibited higher susceptibility to pacing-induced AF and, furthermore, mitochondrial ROS levels were also higher than in wildtype. An association between increased ROS levels and higher prevalence of arrhythmia was suggested by blunting of ROS after crossing the model with another mouse line overexpressing mitochondrial catalase. These mice did not show increased Ca^2+^ leak or increased AF susceptibility, despite the RyR mutation, thus supporting evidence already found in ventricle, that RyR oxidation leads to pro-arrhythmic activity [28, 124]. Therefore, a vicious cycle can develop between ROS and disturbed Ca^2+^ handling, whereby increased SR Ca^2+^ leak could impair mitochondrial function, leading to increased ROS production which triggers further Ca^2+^ leak [124].

#### Open aspects for future research

The exact mechanism by which pro-arrhythmic cytosolic Ca^2+^ dynamics would impair mitochondrial function has yet to be fully resolved. However, we hypothesise that large alterations of cytosolic Ca^2+^, caused by increased Ca^2+^ leak from the SR, or upon defibrillation or during reperfusion after an ischaemic period, may result in a “push” condition at the ETC, as opposed to the aforementioned “pull”. This could increase the production of ^∙^O_2_^−^, thereby changing the redox environment to a state where rate of mitochondrial ROS production exceeds the rate of ROS scavenging, thus resulting in net mitochondrial ROS emission (Fig. 6). A recent study demonstrated that optimising mitochondrial activity pharmacologically with SS31 (a stabiliser of cardiolipin and, therefore, ETC activity) and by pharmacologically preventing increased Ca^2+^ influx can protect against the effects of tachypacing [120]. Initial findings in human also suggested CaMKII oxidation (and thus activation) by ROS as an important link between oxidative stress and AF [102]. As CaMKII can become constitutively active through this mechanism, this may represent another way in which oxidative stress could underlie chronic atrial pathology [36].

A further perpetuating cycle, involving mitochondria and suggested to play a role in myocardial pathologies, is the so-called ROS-induced ROS release (RIRR), as reviewed by Zorov et al. [131]; ROS activate the mPTP, inner membrane anion channel and ATP-sensitive K^+^ channel, thereby dissipating ∆*Ψ*_m_ [7, 126, 127, 130]. The consequence of this is that NADH and NADPH (via reverse NNT) must be used to restore ∆*Ψ*_m_ at the expense of the mitochondrial ROS-scavenging system, thus increasing mitochondrial ROS emission. Furthermore, when open, the mPTP and inner membrane anion channel are permeable to ROS, which can then propagate in the cytosol, potentiating ROS formation and release from neighbouring mitochondria [16, 127]. One would imagine the effect of diffusing ROS is greater in areas of high mitochondrial density and indeed electron microscopic analysis of human atria revealed mitochondrial aggregation in AF [108]. At times of high oxidative stress, for example in heart failure, cross-talk between different ROS sources likely amplifies the ROS signal [73]. Permanent collapse of ∆*Ψ*_m_ induces cell death; however, the metabolic oscillations, caused by increased ROS and RIRR, induce arrhythmia in ventricle [2]. Whether RIRR also underlies pro-arrhythmic activity in the atria during AF is still unresolved; however, this remains an interesting line of investigation.

## Summary and conclusions

The limited understanding of cellular and molecular mechanisms governing AF pathophysiology impedes the development of effective target-specific therapeutic strategies. In the present review, we focussed on the potential role that mitochondria may play in AF. Mitochondrial energetics, Ca^2+^ handling and ROS dynamics all represent major targets for future research, as it is conceivable that the redox environment plays a crucial role during AF. In the current review, we discussed a hypothesis that remodelling and increased energy demand during AF lead to oxidative stress, shifting the redox environment to a state of energy deficit and compromised ROS scavenging capacity (reverse NNT hypothesis), a similar scenario as has been suggested for heart failure [97] (Fig. 6). On the other hand, we also hypothesise that in conditions of “Ca^2+^ overload”, as may occur upon increased intracellular leak, or upon ischaemia reperfusion or defibrillation, mitochondria may experience the other extreme of redox environment, which could increase net ROS emission when ROS production exceeds mitochondrial scavenging capacity (Fig. 6). Therefore, it will be of vital importance in the future to ascertain if and what alterations in Ca^2+^ dynamics—relative to energy demand—occur, how they are associated to remodelling processes in AF (cause vs. effect) and how such changes relate to altered mitochondrial ROS emission. Furthermore, the implementation of the correct experimental environment will be crucial for such investigations, as parameters such as excitation frequency, energy substrate supply and oxygen levels will influence mitochondrial energetics and oxidative stress.

## Electronic supplementary material

Below is the link to the electronic supplementary material.

Supplimentary file (DOCX 15 KB)
