# Supplementary material for: Cellular and mitochondrial mechanisms of atrial fibrillation
Source: Basic Res Cardiol. 2020 Nov 30;115(6):72. doi: 10.1007/s00395-020-00827-7 (PMC7704501; doi:10.1007/s00395-020-00827-7)
Supplement: Supplementary file 1 — Supplimentary file (DOCX 15 KB) [file 395_2020_827_MOESM1_ESM.docx]

**Publications included in Fig 1. (Venn diagram).** Results of Medical Subject Heading (MeSH) search in PubMed. MeSH: “Atrial fibrillation” and “Mitochondria”. List excludes review and editorial publications. Other relevant publications cited in the present manuscript have been added to the list.

**Ad N *et al.*** 2005 J Thorac Cardiovasc Surg 129:41–45.

**Akar JG *et al****.* 2003 Circulation 107:1810–1815.

**Anderson EJ *et al.*** 2014 J Am Heart Assoc 3:e000713.

**Ausma J *et al.*** 2000 Cardiovasc Res 47:788–796.

**Barbey O *et al.*** 2000 J Cardiovasc Electrophysiol 11:432–438.

**Boglepov NN *et al*.** 1974 Zh Nevropatol Psikhiatr Im S S Korsakova 74:1349-1354.

**Bukowska A *et al.*** 2008 Exp Biol Med 233:558–574.

**Chang J-P *et al.*** 2015 Exp Mol Pathol 99:65–73.

**Chen G *et al.*** 2016 Mol Med Rep 14:5311–5317.

**Dong J *et al.*** 2016 J Cardiovasc Pharmacol Ther 21:114–126.

**Emelyanova L *et al.*** 2016 Am J Physiol Circ Physiol 311:H54–H63.

**Goudarzi M *et al.*** 2011 J Proteome Res 10:3484.3492.

**Gunnar RM *et al.*** 1975 Ann N Y Acad Sci 252:264–272.

**Harskamp RE *et al.*** 2019 Am Heart J 211:54–59.

**Jeganathan J *et al.*** 2017 Ann Thorac Surg 104:1547–1555.

**Kalifa J *et al.*** 2008 Mol Cell Biochem 317:69–75.

**Kanaan GN *et al.*** 2019 Can J Diabetes 43:67-75.e1.

**Konishi H *et al.*** 2017 Intern Med 56:1771–1779.

**Laky D *et al.*** 2011 Rom J Morphol Embryol 52:95–98

**Lewis PD *et al.*** 1971 J Neurol Sci 13:381–388.

**Lin P-H *et al.*** 2003 Free Radic Biol Med 35:1310–1318.

**Luo B *et al.*** 2018 Int J Mol Med 42:1125–1133.

**Mary-Rabine L *et al.*** 1983 Circ Res 52:188–199.

**Montaigne D *et al.*** 2013 J Am Coll Cardiol 62:1466–1473.

**Ozcan C *et al.*** 2019 J Card Fail 00:911–920.

**Reilly SN *et al.*** 2011 Circulation 124:1107–1117.

**Sandler N *et al.*** 2018 Hear Lung Circ 27:122–129.

**Schäfler AE *et al.*** 2002 Ann Thorac Surg 74:767–770.

**Shao Q *et al.*** 2019 Cardiovasc Diabetol 18:165.

**Sharma S *et al.*** 2014 Cardiovasc Pathol 23:319–326.

**Slagsvold KH *et al.*** 2014 Physiol Rep 2:e12124.

**Slagsvold KH *et al.*** 2014 Physiol Genomics 46:505–511.

**Soltész B *et al.*** 2019 J Biotechnol 299:66–71.

**Tsuboi M *et al.*** 2001 Eur J Clin Invest 31:489–496.

**Valli H *et al****.* 2017 Mech Ageing Dev 167:30–45.

**Vitadello M *et al.*** 2001 Circulation 103:2201–2206.

**Waymire K *et al.*** 1976 J Cell Physiol 89:345–353.

**Wiersma M *et al.*** 2019 Cells 8:1202.

**Xie W *et al.*** 2015 Sci Rep 5:11427.

**Xu J *et al.*** 2013 J Cardiothorac Surg 8:34.

**Zhang X *et al****.* 2017 J Am Heart Assoc 6:e005945.

**Zou D *et al.*** 2016 Life Sci 156:7–14.
